# Emergency Department visits due to intoxications in a Dutch university hospital: Occurrence, characteristics and health care costs

**DOI:** 10.1371/journal.pone.0226029

**Published:** 2019-12-19

**Authors:** C. Verheij, P. P. M. Rood, C. K. Deelstra, M. L. L. Levendag, B. C. P. Koch, S. Polinder, S. C. E. Schuit, J. A. Haagsma

**Affiliations:** 1 Department of Emergency Medicine, Erasmus MC, University Medical Center Rotterdam, Rotterdam, The Netherlands; 2 Department of Pharmacy, Erasmus MC, University Medical Center Rotterdam, Rotterdam, The Netherlands; 3 Department of Public Health, Erasmus MC, University Medical Center Rotterdam, Rotterdam, The Netherlands; 4 Department of Internal Medicine, Erasmus MC, University Medical Center Rotterdam, Rotterdam, The Netherlands; Medical University Graz, AUSTRIA

## Abstract

**Background:**

Intoxications with alcohol and drugs are common in the Emergency Department. This study aimed to describe the occurrence and characteristics of intoxications (alcohol, Drugs of Abuse (DOA), pharmaceutical and chemical) presented to the Emergency Department and the health care costs of these intoxications.

**Methods:**

This was a retrospective medical record study of all patients (≥ 16 years) who presented to the Emergency Department of an inner-city academic hospital in the Netherlands due to single or multiple intoxication(s) as the primary *or* secondary reason in the year 2016. An intoxication was reported as present if the attending physician described the intoxication in the patient’s medical record.

**Results:**

A total of 783 patients were included, accounting for 3.2% of the adult Emergency Department population (age ≥ 16 year). In 30% more than one substance was used. Intoxications with alcohol, Drugs of Abuse and pharmaceuticals was reported in respectively 62%, 29% and 21% of the intoxicated patients. The mean costs per patient presenting with an intoxication to the Emergency Department was € 1,490. The mean costs per patient were highest for pharmaceutical intoxications (€ 2,980), followed by Drugs of Abuse (€ 1,140) and alcohol (€ 1,070).

**Conclusions:**

Intoxications among patients aged 16 years and older are frequently seen at the Emergency Department and are frequently caused by multiple substances. Alcohol is the most common intoxication. Substantial healthcare costs are involved. Therefore, this study suggests that further research into hazardous alcohol consumption and DOA abuse is warranted.

## Introduction

Intoxications with alcohol and drugs are a common problem in the Emergency Department (ED). In the literature approximately 1–5% of all visits to EDs are due to single or multiple intoxication(s).[1,2]

Intoxications vary from alcohol, drugs (pharmaceutical, non-pharmaceutical or illicit drug use/drugs of abuse (DOA) to carbon monoxide (CO) and chemicals.[1–3] Globally, alcohol intoxication is the most prevalent intoxication among patients presenting to the ED.[2,4–9] Over time, changes in the number of specific types of intoxications are seen in other countries.[10–16] There is an increase in alcohol related visits to the ED in the United States (US).[10,11] Also, an increasing trend in the occurrence of (different types of) DOA related attendances is seen, although not all studies support these results.[11–13] Among pharmaceuticals, an increase in ED visits concerning benzodiazepines and opioids is reported up to 200% in the United States.[12,14–16] No recent data is available about the occurrence and characteristics due to intoxications in the ED in the Netherlands.

Intoxicated patients are considered to be a burden on healthcare worldwide. This burden includes social and economic aspects as well as the burden placed on hospitals.[17–21] The increase in ED visits and hospital admissions due to intoxications has been described in literature. The subsequent use of healthcare resources is part of a growing burden on our healthcare system.[20] However, no recent data is available on health care consumption due to intoxications in the ED in the Netherlands.

Therefore, the current study aimed to describe the occurrence and characteristics of intoxications presented to the ED of an inner-city academic hospital in the Netherlands. Second, this study aimed to describe the health care costs of these intoxications.

## Materials and methods

This was a retrospective medical record study. The ethics committee approved this study (MEC-2016-629). No informed consent was needed because of the retrospective, observational design of this study.

The study population consisted of all patients aged 16 years and older who needed medical care at the ED of the Erasmus MC University Medical Center, an inner-city academic hospital in the Netherlands, due to a single or multiple intoxication(s) in the year 2016. Intoxication was defined as a condition that follows the administration of a substance and results in disturbances in the level of consciousness, cognition, perception, judgement, affect, or behavior, or other (psycho-) physiological functions and responses.[22] Intoxications included alcohol, drugs (pharmaceutical, non-pharmaceutical or DOA), carbon monoxide (CO) and chemicals. For this study, an intoxication was reported as present if the attending physician described the intoxication in the patient’s medical record. Inclusion criteria were all patients aged ≥ 16 years presenting to the ED between January 1, 2016 and December 31, 2016 who either presented to the ED with an intoxication as the primary *or* secondary reason. A secondary reason was defined as presentation to the ED for a reason other than intoxication, but upon examination it became clear that medical care for a present intoxication was needed.

Researchers retrieved a range of variables from the electronic medical records. Variables included patient characteristics (gender, age, intentional versus nonintentional exposure), characteristics of the intoxications (type, co-intoxications), variables regarding therapy, diagnostics and health care consumption due to intoxications (ambulance transportation, length of stay at the ED, day and time of presentation, admittance to ward or Intensive Care Unit (ICU) including length of stay) between ED visit and discharge.

With regard to the health care costs, our study focused on the direct, pre-hospital and in-hospital care during treatment of intoxication. We considered costs of the ED visit, in-hospital stay at a general ward and ICU and costs of ambulance transportation. Non-medical costs, for example productivity loss, were not considered. Unit costs were retrieved from the cost-reference manual provided by the Dutch National Health Care Institute.[23,24] Mean costs for a visit to the ED are € 259. The rate of ambulance transport is € 613. The mean costs for one day admittance to the ward in an academic and general hospital are €642 and € 443, respectively. The average day costs of ICU (including diagnostics and medication) admission was valued at € 2,015.[23] Health care use per period was determined and multiplied with the costs per unit. When admitted, a proportion of the patients was transferred to regional (general) hospitals instead of admittance to the Erasmus MC University Medical Center.

The length of stay when patients were transferred to a general hospital was not known; for the cost calculation we assumed that the length of stay in a general hospital was similar to the length of stay in the Erasmus MC University Medical Center.

Subgroups of different intoxications were compared to see whether these groups differed in costs.

### Statistical analysis

The data were extracted to and analyzed using SPSS (IBM Corp. Released 2016. IBM SPSS Statistics for Windows, Version 24.0. Armonk, NY: IBM Corp.). The data were analyzed using descriptive statistics; mean, median, standard deviation and proportions were described. We assessed the distribution of age, gender, DOA, time of presentation to the ED, length of hospital stay, discharge category and health care costs for all patients, as well as by intoxication category. We have used the Shapiro-Wilk test to test for the normality of data. Any missing data were described.

## Results

In 2016, a total of 24,720 patients (age ≥ 16 year) were seen in the ED. A total of 783 patients met the inclusion criteria for this study, accounting for 3.2% of the total ED population. In 30% of all intoxications more than one substance was used.

### Characteristics of patients presenting with intoxication

The mean age of patients presenting with intoxication was 35 years (range 16–88; standard deviation (SD) 15.2). Of all patients presenting with an intoxication, 67% (n = 523) was male. The socio-demographic details of all patients that presented with an intoxication as well as by category of intoxication are shown in Table 1. Almost half of all patients (47%) had a maximum Glasgow Coma Score (GCS). In almost 3% (n = 23) of patients the GCS score was 3 and 3% (n = 27) was intubated. Almost 16% (n = 119) of patients had previously visited the ED with an intoxication. In 17% (n = 133) of the patients the intoxication was an attempted suicide. Pharmaceutical drugs were involved in 85% (n = 113) of all suicide attempts, whereas DOA and alcohol were involved in 15% (n = 20) and 27% (n = 36), respectively. One patient died during admittance at the Erasmus MC University Medical Center. No patients died during their stay in the ED.

**Table 1:** Socio-demographics of patients presenting to the emergency department with intoxications.

	n	Gender (% male)	Age (mean; range; SD)
**Total group of intoxications**	783	67%	35; 16–88; 15.2
**Alcohol**^**a**^	484	70%	34; 16–79; 14.9
**DOA**^**a**^	227	75%	31; 16–77; 10.9
**Pharmaceuticals**^**a**^	162	49%	43; 16–88; 15.3
**Chemicals**^**a**^	23	83%	37; 23–76; 14.04

^a^Please note that some patients may be categorized into more than one category due to combination intoxications, e.g. patients with an alcohol intoxication can also have a intoxication with drugs of abuse (DOA).

### Characteristics of intoxications

**Fig 1:**
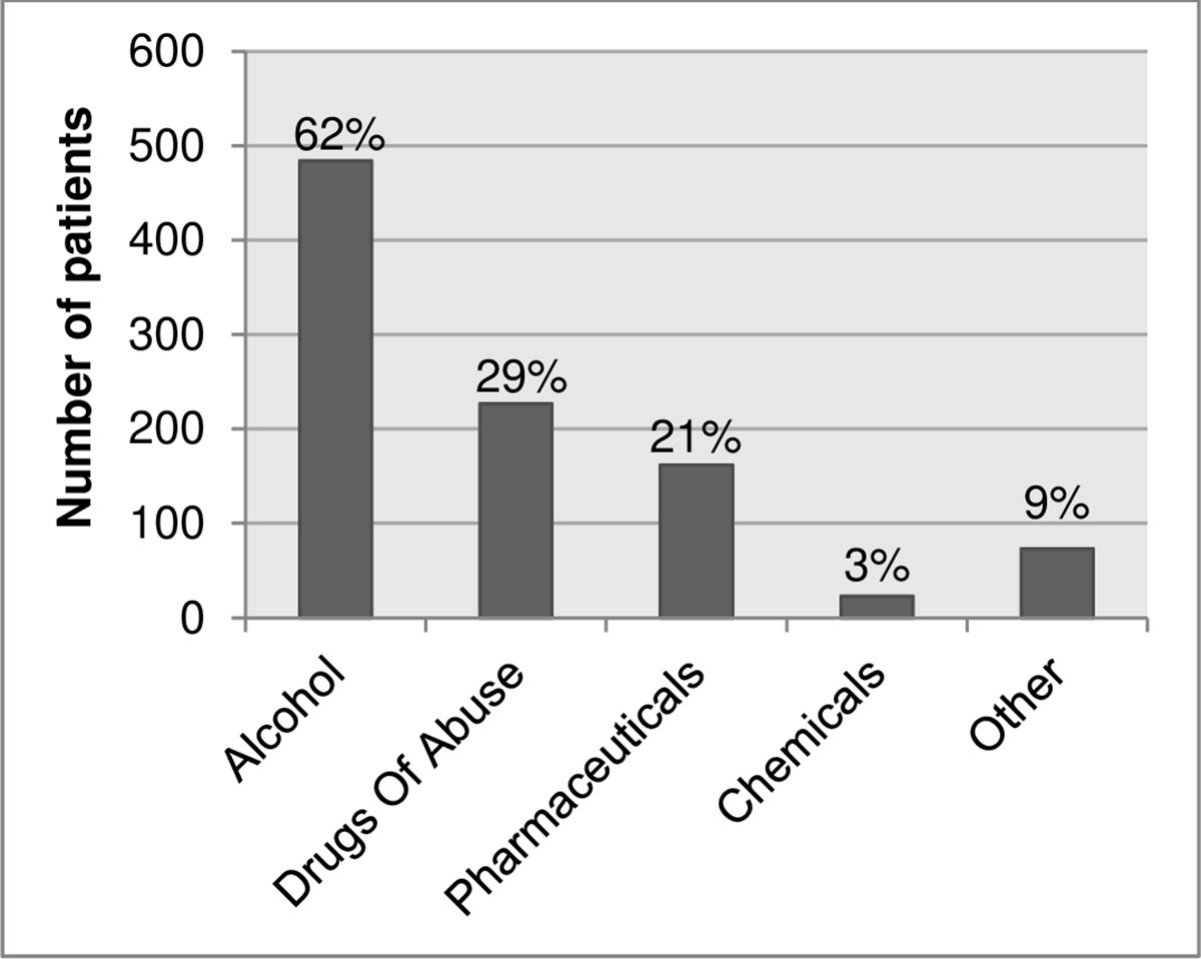
Distribution of different types of intoxications seen at the emergency department.

The distribution of the different types of intoxications is shown in Fig 1.

#### Alcohol

Of the total group of 783 patients, in 62% (n = 484) alcohol was the (co-)intoxicant. The mean age of ED patients with an alcohol intoxication was 34 years (range: 16–79; SD 14.9) and 70% (n = 339) was male. In 31% (n = 150) of all intoxications with alcohol a co-intoxication was present; these were mostly co-intoxications with DOA (n = 109) or pharmaceuticals (n = 37). A total of 7 (1%) patients had a combined intoxication with alcohol, DOA and pharmaceuticals.

#### Drugs of abuse

**Fig 2:**
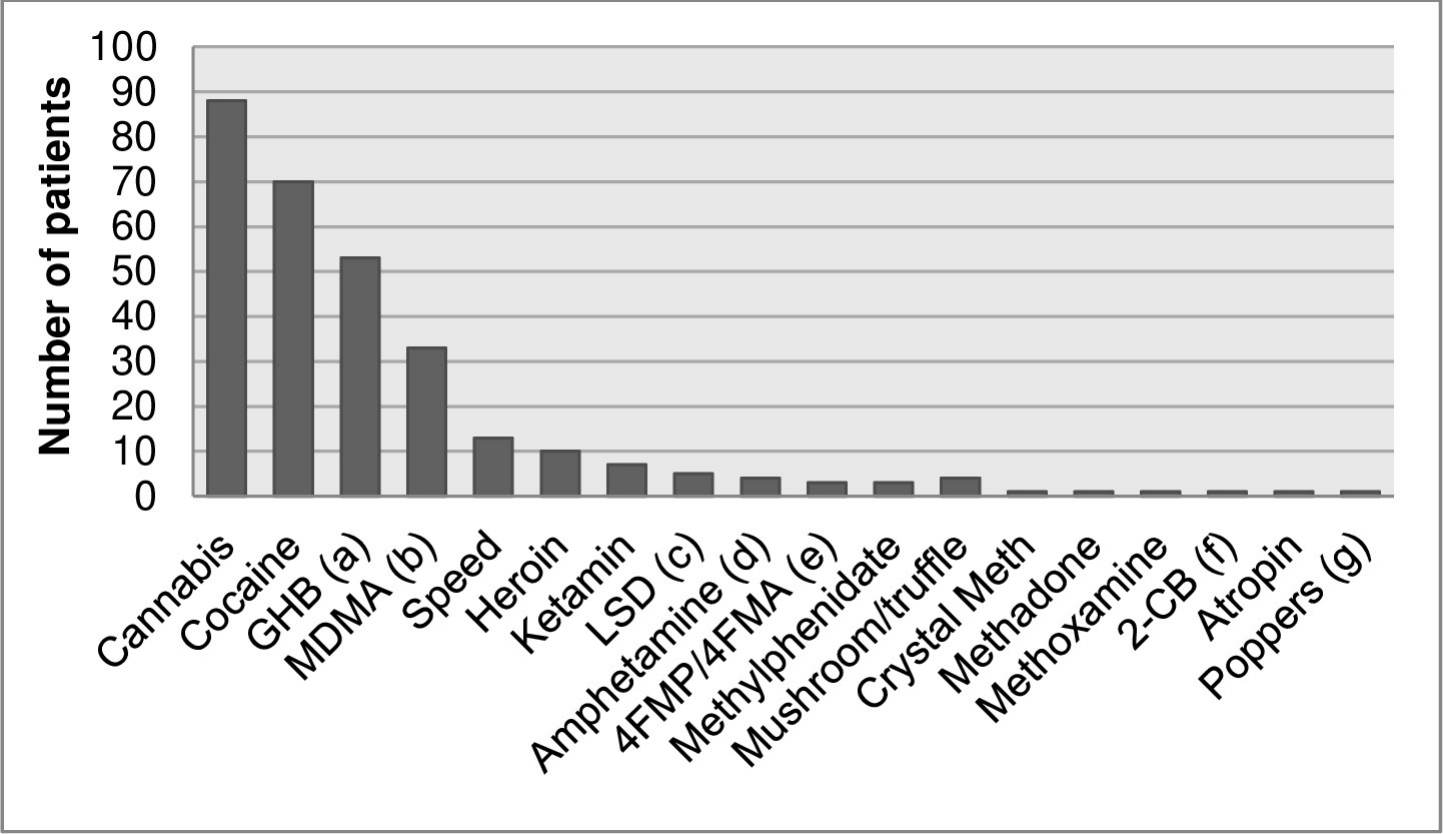
Number of intoxications with drugs of abuse seen at the emergency department, by type of drugs of abuse (n = 227). (a) gammahydroxybutyrate. (b) 3,4-methylenedioxymethamphetamine. (c) lysergic acid diethylamide. (d) amphetamine of unknown type. (e) 4-fluoroamphetamine. (f) 2,5-dimethoxy-4-bromophenethylamine. (g) alkyl nitrites.

DOA caused 29% (n = 227) of all intoxications. The mean age of people with an intoxication with DOA was 31 years (range: 16–77; SD 10.9) and 75% (n = 170) of the patients were male. In 48% (n = 109) of these patients, a co-intoxication with alcohol was present. Combination of DOA and pharmaceuticals was seen in 11% (n = 25) of all drugs of abuse cases. The most frequently seen DOA intoxications were cannabis (n = 88; 11% of all intoxications and 39% of all DOA intoxications), cocaine (n = 70; 9% of all intoxications and 31% of all DOA intoxications), gammahydroxybutyrate (GHB) (n = 53; 7% of all intoxications and 23% of all DOA intoxications) and 3,4-methylenedioxymethamphetamine (MDMA/ecstasy) (n = 33; 4% of all intoxications and 15% of all DOA intoxications), respectively. These four intoxications together accounted for 244 intoxications in 202 patients because some people were intoxicated with more than one DOA. The number of cases of other groups of DOA are shown in Fig 2.

#### Pharmaceutical drugs

**Fig 3:**
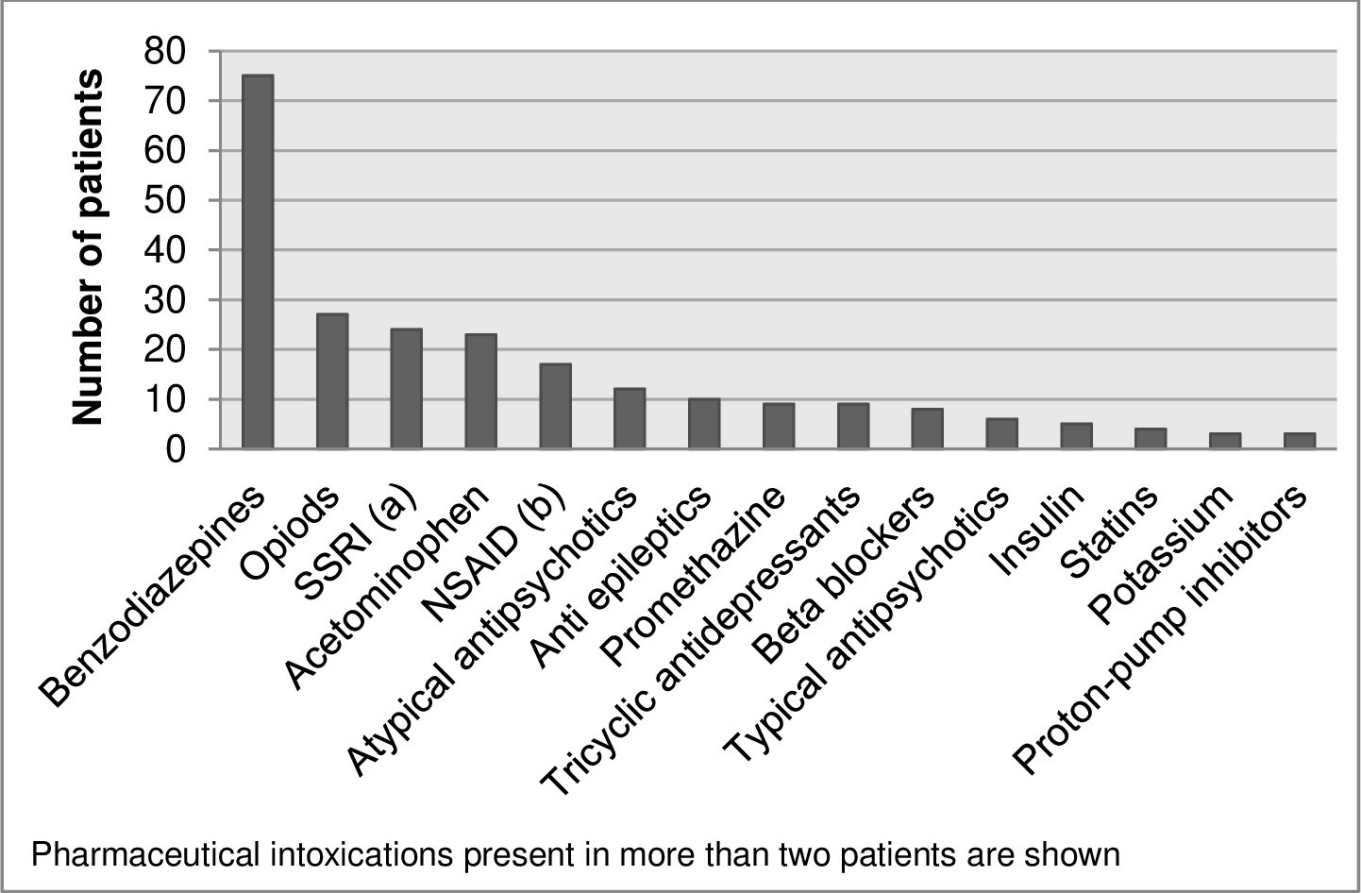
Number of intoxications with pharmaceutical drugs seen at the emergency department, by type of pharmaceutical. (a) selective serotonin reuptake inhibitors. (b) non-steroidal anti-inflammatory drugs.

Intoxication with pharmaceutical drugs was present in 21% (n = 162) of patients. The mean age of patients with an intoxication with pharmaceutical drugs was 43 years (range: 16–88; SD 15.3) and 49% (n = 79) was male. In 23% (n = 37) of these patients, a co-intoxication with alcohol was present. Almost half of the patients had taken benzodiazepines (n = 75; 10% of all intoxications and 46% of all pharmaceutical drug intoxications). Opioids were seen in 17% (n = 27) of all pharmaceutical drug intoxications (3% of all intoxications) followed by selective serotonin-reuptake inhibitors (SSRI) in 15% (n = 24) of all pharmaceutical drug intoxications (3% of all intoxications), acetaminophen in 14% (n = 23, 3% of all intoxications) and non-steroidal anti-inflammatory drugs (NSAID) in 10% (n = 17, 2% of all intoxications). Pharmaceutical drugs present in more than two patients are shown in Fig 3. In addition, 32 other pharmaceutical drugs were found in 1 or 2 patients. Multiple pharmaceutical drugs were taken in 67 (41%) of all pharmaceutical drug intoxications.

#### Chemicals

In 3% of all intoxications, chemicals were involved; mostly propane gas (n = 4), 1,2-dichlorethane (n = 3) and toluene (n = 4). Also intoxications with petroleum (-vapor) (n = 2), aniline (n = 1), carbon dioxide absorption granules (n = 1), hydrogen sulfide (n = 1), arsenic (n = 1), methylated spirit (n = 1), chlorine (n = 2), glacial acetic acid (n = 1), potassium permanganate (n = 1), isopropyl alcohol (n = 1) and mouse poison (n = 1).

#### Other intoxications

In the total group of patients, there were 47 intoxications other than alcohol, DOA, pharmaceuticals or chemicals. Most of these patients had an intoxication with CO (n = 36) and three patients presented with complaints after the use of Nitrous Oxide (N_2_O). The other intoxications included in this category were intoxications (all single cases) with cyanide, snuff tobacco, water, helium and intoxications with unknown composition (shampoo, water pipe smoking, denture cleanser, herbal pills). In 28 patients (4% of all intoxications) the presence of an intoxication was described in the medical record, but there was no information available about the exact type of intoxication.

### Presentation to the ED and health care consumption

Most intoxicated patients presented at the ED on Friday (18%, n = 138), Saturday (20%, n = 156) or Sunday (22%, n = 169). Almost half (44%) of the patients came in during nighttime hours (midnight– 6 AM) and more than a quarter (27%) during evening hours (6 PM—midnight). In 75% of all intoxications some form of diagnostics was performed, mostly general blood samples (54%) and ECGs (41%). Specific medication concentration measurement in blood or DOA screening in urine was performed in 19% and 6%, respectively. Radiologic exams were performed in 34% of all patients; mostly CT-cerebrum (26% of all intoxications). An antidote was given in the ED in 5% of all intoxications (mostly naloxone; 3% of all intoxications) and in 1% it was stated in the medical record that an antidote was given pre-hospital. Gastric lavage took place in four patients (1%) and active charcoal was administered in 3% of all patients. Medication other than an antidote was given in 38% of the patients, including benzodiazepines which were administered in 4.3%. Three percent of patients were intubated. The mean length of stay of all intoxications at the ED was 3:55 hours (range: 0:12–22:03; SD 2:20). Twenty-five percent of all intoxications was admitted to the hospital, of whom 15% to the ICU. The remaining patients were discharged home (66%), to a psychiatric, addiction or homeless center (4%), to the police station (1%) or left against medical advice (4%). In the group presenting with an alcohol intoxication, 20% was admitted. In the group with a DOA intoxication 16% was admitted. In the group with a pharmaceuticals intoxication 52% was admitted. Of all admitted patients, most patients were admitted to the Internal Medicine ward (24%) or Neurology ward (22%); 15% of the admitted patients were admitted to the ICU, accounting for 4% of the total group of intoxications. Almost one quarter (24%) of all admitted patients were transferred to another hospital; more than half of these were admittances to the ICU (55%). The mean length of stay in the ICU of the Erasmus MC University Medical Center was 4.0 days (range 1–12; SD 4.5). The mean length of stay in the ward was 3.4 days (range 1–43; SD 5.7). The mean length of stay was highest for intoxications with pharmaceuticals (5.5 days; range 1–43 days; SD 7.7) and DOA (3.6 days; range 1–17; SD 5.1). The mean length of stay, when admitted, if alcohol was involved was 2.8 days (range 1–24; SD 4.7).

### Healthcare costs

The total mean costs per patient presenting with an intoxication at the ED were € 1,490 when combining the estimated costs for ambulance transportation, ED visits and hospital admission. The mean costs per patient are highest for pharmaceutical intoxications (€ 2,980) followed by DOA (€ 1,140) and alcohol (€ 1,070); see also Table 2.

**Table 2:** Estimation of total costs of intoxications and per cost component.

Intoxication	n	Costs ED presentation (€)	Presentation with ambulance (n)	Costs Ambulance (€)	Admittance academic hospital (n)^b^	Admittance general hospital (n)^b^	Length of stay during admission (days)^b^	Costs of admittance^c^ (€)	Mean costs per patient (€)	Total costs (€)
**Total group of intoxications**	783	202,800	616	377,610	149	46		585,720	**1,490**^d^	1,166,130^d^
					ICU: 5	ICU: 25	ICU: Mean 4.00; range 1–12; SD 4.5	AH: 352,770		
					Ward: 144	Ward: 21	Ward: Mean 3.38: range 1–43; SD 5.7	GH: 232,940		
**Alcohol^a^**	484	125,360	411	251,940	85	12		141,380	**1,070**	518,680
					ICU: 1	ICU: 6	ICU: Mean 2	AH: 111,890		
					Ward: 84	Ward: 6	Ward: Mean 2; range 1–27; SD 4.8	GH:29,500		
**DOA^a^**	227	58,790	173	106,050	21	15		94,800	**1,140**	259,640
					ICU: 2	ICU: 13	ICU: Mean 1.50; range 1–2; SD 0.7	AH: 52,150		
					Ward: 19	Ward: 2	Ward: Mean 3.78; range 1–17; SD 5.3	GH:42,640		
**Pharmaceuticals^a^**	162	41,960	111	68,040	59	25		373,010	**2,980**	483,010
					ICU: 4	ICU: 12	ICU: Mean 4.5; range 1–12; SD 5.1	AH: 232,240		
					Ward: 55	Ward: 13	Ward: Mean 5.55; range 1–43; SD 7.9	GH: 140,770		

^a^Please note that some patients may be categorized into more than one category due to combination intoxications, e.g. patients with an alcohol intoxication can also have a intoxication with DOA.

^b^ ICU: Intensive Care Unit

^c^ AH: Academic Hospital; GH: General Hospital.

^d^ Please note that the numbers below don’t add up because of combination intoxications.

The total annual costs for patients presenting with intoxications at the ED of the Erasmus MC University Medical Center were € 933,180 when combining the estimated costs for ambulance transportation, ED visits and the costs of hospital admission. Adding the extrapolated costs for admittance of transferred patients in other (general) hospitals results in total costs of € 1,166,130. Alcohol intoxication (as mono- or combination intoxication) accounted for almost half (45%) of the total costs, followed by (mono- or combination) intoxications with pharmaceuticals (41%) and DOA (22%); see also Table 2.

Half of the total costs was accounted for by the costs of hospital admission. The costs of presentation to the ED accounted for 18% and the costs of ambulance transportation accounted for 32% of the total costs; see also Table 2.

## Discussion

The current study showed that intoxications among patients aged 16 years and older are frequently seen in the ED accounting for 3.2% of all ED visits at Erasmus MC University Medical Center. The most frequently seen intoxication was alcohol which was present in the majority (62%) of all patients presenting with an intoxication. Intoxications with DOA and pharmaceuticals accounted for respectively 29% and 21% of the study population. The mean costs per patient of patients presenting with an intoxication at the ED was € 1,490. The mean costs per patient was highest for pharmaceutical intoxications. The biggest proportion (45%) of the total costs was due to alcohol intoxications as a result of the large number of patients presenting to the ED with this type of intoxication.

The occurrence of all intoxications treated in the ED found in this study is higher than those found in literature in the Netherlands.[1,4] Possible explanations could be a higher alcohol and/or drug use in the population over time, a higher use of alcohol and/or drugs in the population in Rotterdam, and/or the inner city hospital location of this study.

In accordance to literature, alcohol was the most prevalent intoxication in our study.[2,4–9] In the current study, a co-intoxication was present in almost one third of all alcohol intoxicated patients. This is higher compared to other studies where alcohol is more often seen as a mono intoxication, although higher rates of combination intoxications are reported.[1,2,4,9] Nevertheless, alcohol was seen in the majority of all intoxications. In literature an increase in alcohol related visits to the ED is described.[10,11] Vermes et al. conducted a similar descriptive study in the Erasmus MC University Medical Center in the year 2000 using a similar methodology.[1] Comparison of the current study with the study by Vermes et al. showed that the reported number of intoxications with alcohol, DOA or pharmaceuticals increased by 38% from 2000 to 2016. In this period, the population of Rotterdam increased by 6.2%.[25] Alcohol was present as a mono- or a combination intoxication with pharmaceuticals and/or DOA in 319 patients in the year 2000 (61% of all alcohol, DOA and/or pharmaceuticals intoxications) compared to 473 patients (66% of all alcohol, DOA and/or pharmaceuticals intoxications) in the current study. The largest difference is seen among mixed intoxications; in 2000 alcohol was present in 41% (n = 42) of all combination intoxications, in 2016 this rate was 60% (n = 139).[1] This increase is alarming and this study suggests that further research into hazardous alcohol consumption is warranted.

Our results regarding the prevalence of DOA and pharmaceutical intoxications is in line with literature which shows that approximately 0.3–2% of all ED visits are drug related.[1,2,26] In the current study cannabis, cocaine, GHB and MDMA were the most frequently seen DOA; this is also seen in literature although our study found lower rates for heroin.[2,4,12,26–28] This is probably due to a low overall prevalence of heroin use in the Netherlands.[29] The most frequently seen DOA was cannabis. This may be explained by the current toleration policy by the Dutch government which implies that people will not be prosecuted for possession or use of small quantities of soft drugs and that the sale of soft drugs in ‘coffee shops’ is tolerated.[30]

The current study shows an increase in the absolute number of intoxications with DOA when comparing the results with the study of Vermes et al. (2003); from n = 96 in 2000 to n = 227 in 2016.[1] This is in line with several studies that show a trend of increasing prevalence of (different types of) drug related ED visits. Cocaine, GHB, ketamine and cannabis are reported to be more frequently observed among ED attendances compared to before, although not all studies support these results.[11–13,31] The alarming increase seen in the current study is in line with the upward trend that is seen in the general population in the Netherlands for the use of cannabis, cocaine, MDMA, amphetamine and GHB.[29]

Our study showed that an intoxication with pharmaceuticals was present in 21% of all patients presenting with an intoxication. Intoxications with benzodiazepines and opioids were most frequently seen, followed by intoxications with SSRI’s, acetaminophen ad NSAID’s. This is in line with previous studies that showed that anti-anxiety and sedative medication (especially benzodiazepines), analgesics (such as acetaminophen and opioids), antidepressants and antipsychotics are the most frequently seen intoxications.[1,4,12,26] The high rate of the current study for benzodiazepine and opioid intoxications is also in line with international literature.[1,4,12,26] Comparison with 16 years ago showed no big differences in the amount and types of pharmaceutical intoxications.[1]

The rates of patients receiving therapy, with low rates for patients receiving antidotes or gastric lavage, were lower compared to rates found in the Dutch study of Ambrosius et al. (2012).[4] A study among Dutch hospitals showed that the use of antidotes and gastric lavage vary among hospitals in the Netherlands and are mostly used in intoxications with pharmaceutical drugs.[32] The relatively small group of these intoxications in the current study could be a possible explanation for the low rates found. Also, gastric lavage is only performed if the patient presents to the ED within the timeframe were this has merit and if the severity of the intoxication outweighs the potential risk of this intervention. In most cases of alcohol and DOA intoxications (which is by far the largest group within this study) there is no merit of this intervention. Also, for most of the intoxications seen in the current study, there is no suitable antidote.

Most patients were seen during the weekends. This is also frequently described in literature.[19–21,33–34] In the current study a quarter of all patients were admitted which is comparable to rates in literature although rates vary widely from 25–78.3%.[1,4,12,26,32] This rate was also comparable to the admittance rate reported by Vermes et al. in 2000 in the Erasmus MC University Medical Center (33%).[1]

The relatively high proportion of admittance after intoxications demonstrates a large burden on healthcare, which is in line with previous studies.[1,4,12,19,20,26,32] In comparison to literature, the mean costs for an ED visit (€ 259) are somewhat lower in comparison to costs seen in literature, where rates vary from £249 among alcohol related attendances (UK) to €541 among attendances due to alcohol intoxications (Belgium) and € 873 among deliberate self-poisoning patients (Belgium).[6,33,35] The differences between the costs can (partly) be explained by different ways of calculation of these costs and differences in costs among countries.[6,33,35] To our knowledge, there are no Dutch studies published on this particular subject. Multiple studies have described that intoxication related attendances to the ED and related hospital admission are part of a growing burden on our healthcare system.[19–20] In comparison with the study of Vermes (conducted 16 years ago in the Erasmus MC University Medical Center), this study also shows a slight increase in the number of intoxications with alcohol, DOA or pharmaceuticals from 520 (2.2% of total adult ED population) to 720 (3.1% of total adult ED population) intoxications.[1]

A possible limitation of the current study is that the majority of the presented data were not obtained from toxicological analysis but from electronic medical records. Therefore, the presented numbers could be an under- or overestimation of the actual occurrence of intoxications at the ED.

Also, for a minority of all transferred patients (24%; 6% of all patients) the length of stay in the general hospitals was based on estimation, since we assumed that length of stay at a ward of a general hospital was similar to the length of stay in the Erasmus MC University Medical Center. Furthermore, the mortality rate of the patients who were transferred to a general hospital was not known. The presented costs are likely to be underestimated because the costs of transportation when patients were transferred to a general hospital were not included since the exact type (ambulance or Medical Intensive Care Unit transport) was unknown.

Finally, it is important to note that this is a single-center study which was conducted in the Netherlands and therefore generalizability to other hospitals in the Netherlands and beyond may be limited. We therefore recommend to establish a nationwide database, or preferably an international database, to investigate regional differences in the occurrence, characteristics and health care costs of intoxications.

## Conclusions

Intoxications among patients aged 16 years and older were frequently seen at the ED of a large inner city tertiary hospital, accounting for 3.2% of all patients. In a third of intoxications, more than one substance was used. Intoxication with alcohol was seen most frequently followed by DOA.

The mean costs per patient for patients presenting with an intoxication at the ED was € 1,490. The mean costs per patient was highest for pharmaceutical intoxications, although the biggest proportion (45%) of the total costs was accounted for by alcohol intoxications due to the large number of patients presenting to the ED with this type of intoxication. Comparison over a 16 year period showed an alarming increase in the absolute and relative numbers of patients presenting with an alcohol and/or DOA intoxication, especially alcohol in combination with other intoxications.

Therefore, this study suggests further research into hazardous alcohol consumption and DOA abuse.
